# A Personalized Approach for Patients with Myocardial Infarction with Non-Obstructive Coronary Arteries

**DOI:** 10.31083/j.rcm2502047

**Published:** 2024-01-29

**Authors:** Leonardo De Luca, Federico Andreoli, Raffaella Mistrulli, Giulia Mattaroccia, Gianmarco Gargano, Domenico Gabrielli

**Affiliations:** ^1^Department of Cardio-Thoracic and Vascular Medicine and Surgery, Division of Cardiology, A.O. San Camillo-Forlanini, 00152 Rome, Italy

**Keywords:** myocardial infarction with non-obstructive coronary arteries, acute coronary syndrome, spontaneous coronary artery dissection

## Abstract

Myocardial infarction with non-obstructive coronary arteries (MINOCA) includes coronary embolism, dissection, spasm and microvascular dysfunction, as well as plaque rupture or erosion (causing <50% stenosis). In the most recent studies, events that can be classified as MINOCA account for approximately 6–8% of all diagnoses of acute myocardial infarction (AMI). Clinical suspect may suggest the need for additional diagnostic procedures beyond the usual coronary angiography, such as cardiac imaging or provocative tests. Cardiac magnetic resonance (CMR) is essential for both validating the diagnosis and ruling out other conditions with a comparable clinical presentation. The prognosis is not as good as previously believed; rather, it is marked by morbidity and mortality rates comparable to those of other types of AMI. Identification of the underlying causes of MINOCA is recommended by current guidelines and consensus documents in order to optimize treatment, enhance prognosis, and encourage prevention of recurrent myocardial infarction. In this narrative review, we have outlined the various causes of MINOCA and their specific therapies in an attempt to identify a personalized approach to its treatment.

## 1. Introduction

Myocardial infarction with non-obstructive coronary arteries (MINOCA) was first described over 80 years ago. In clinical practice, the term has been widely and inconsistently applied, influencing various aspects of disease classification, investigation, and management.

The European Society of Cardiology (ESC) position statement on MINOCA proposed the following criteria: (1) acute myocardial infarction (AMI) criteria as defined by the ‘Third universal definition of myocardial infarction’; (2) non-obstructive coronary arteries, with no lesions ≥50% in a major epicardial vessel and (3) no other clinically overt specific cause that can serve an alternative cause for the acute presentation [1].

Essential for the definition of MINOCA is the diagnosis of AMI with an elevated cardiac biomarker. However, the increase in troponin levels is non-specific and can result from either ischemic or nonischemic mechanisms. Thus, the term MINOCA should be reserved for patients in whom there is an ischemic basis for their clinical presentation [2].

MINOCA has several different pathophysiological pathways, including coronary embolism, dissection, and spasm, as well as plaque rupture or erosion [3, 4].

**Fig. 1.**
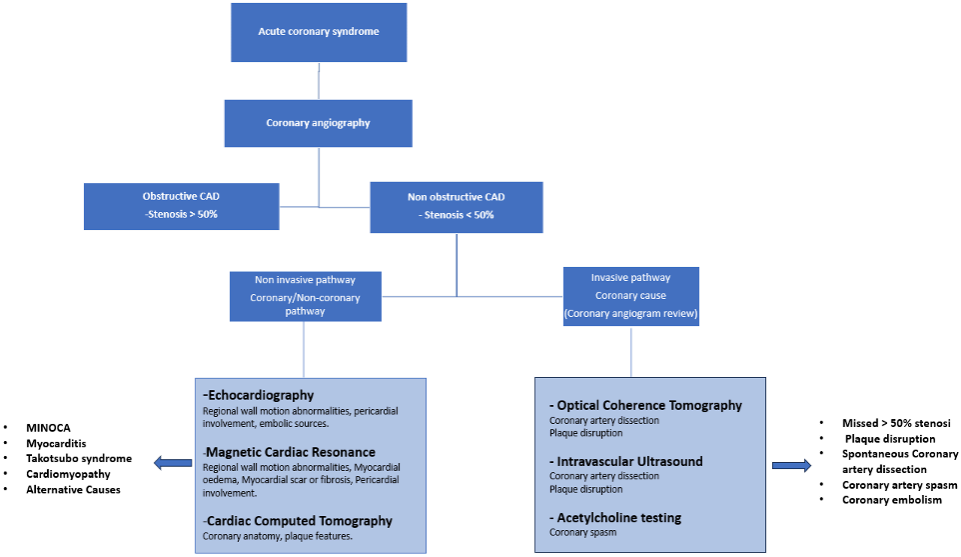
**Diagnostic algorithm of acute coronary syndrome without obstructive CAD**. CAD, coronary artery disease; MINOCA, myocardial infarction with non-obstructive coronary arteries.

According to the ‘Fourth Universal Definition of Myocardial Infarction’, it is possible to classify AMI into 5 types, depending on the underlying mechanism. MINOCA cases account for about 5–20% of type 1 AMIs (characterized by spontaneous intracoronary obstruction, even if not detectable at the time of coronarography) and a large proportion of type 2 AMIs (where the mechanism is the discrepancy between oxygen demand and oxygen supply to the myocardium) [5]. In the most recent studies, events that can be classified as MINOCA account for approximately 6–8% of all diagnoses of AMI [6]. Compared to the population of subjects with AMI and obstructive coronary artery disease (AMI-CAD), MINOCA patients are generally younger, with a mean age at presentation of about 55 years and only a slight preponderance of the male sex [2]. The female sex is therefore proportionally more represented than its AMI-CAD counterpart, with values of around 40%, while in the particular case of coronary artery dissections, the female population is the most affected sex. The cardiovascular risk factor profile of MINOCA patients does not differ substantially from the AMI-CAD population, except for a lower prevalence of dyslipidaemia and diabetes [2, 4]. At the time of hospital presentation, about two thirds of MINOCA patients present with an electrocardiographic pattern that can be classified as an AMI in the absence of ST-segment elevation (NSTEMI), while in the remaining third the presentation is that of myocardial infarction with ST-segment elevation (STEMI) [5]. Considering the multifactorial origin of MINOCA, clinical suspicion should dictate the need for additional diagnostic procedures beyond the usual coronary angiography, such as intravascular ultrasound (IVUS), optical coherence tomography (OCT), invasive provocative testing for vasospasm, testing for hypercoagulable disorders, and CMR. CMR is one of the key diagnostic tools in this algorithm for the differential diagnosis of Takotsubo syndrome, myocarditis, or true AMI. CMR has the ability to identify the underlying cause in as many as 87% of patients with MINOCA [7, 8]. Intracoronary acetylcholine or ergonovine testing may be performed when coronary or microvascular spasm is suspected (Fig. 1). However, despite optimal work-up, the cause of MINOCA remains undetermined in 8–25% of patients. Although associated with better prognosis compared to patients with ACS patients with AMI-CAD, MINOCA patients have a lower survival rate than healthy individuals matched for age and sex [9]. Of importance, this excess of adverse events has been reported at both early and late follow-up.

The mortality rate from all causes at 12 months ranges from 2% to 4.7% [10, 11, 12]. Finally, in MINOCA patients, long-term quality of life also appears to be impaired: persistence of angina symptoms at 1 year has been documented in 25% of cases [9]. In this narrative review of the literature, we discuss the pathophysiology and management of MINOCA according to the latest evidence.

## 2. Pathophysiology of MINOCA

### 2.1 Coronary Atherosclerotic Causes of MINOCA

Plaque disruption is a common cause of MINOCA, which includes plaque rupture, plaque erosion, and calcific nodules. Plaque disruption could cause thrombus formation, leading to AMI by distal embolization or superimposed coronary spasm; and after fibrous cap disintegration, the highly thrombogenic plaque core is suddenly exposed to the flowing blood. This condition could generate complete transient thrombosis with spontaneous thrombolysis causing MINOCA [13]. The risk of plaque disruption is related to intrinsic properties of individual plaques (*plaque vulnerability*) and extrinsic forces acting on plaques (*rupture triggers*) [13]. The former predisposes plaques to rupture, whereas the latter may precipitate disruption of vulnerable plaques [14, 15]. According to Ouldzein *et al*. [16] the rate of ruptured plaques among 68 MINOCA patients was nearly 37%. The prevalence of plaque rupture could be even higher with more extensive use of higher-resolution imaging (es OCT), since other methods such as IVUS do not recognize plaque erosion [17]. Plaque erosion is the second most common cause of atherothrombosis (30–35%) [18, 19, 20, 21, 22]. Plaque erosion and plaque rupture are different phenotypes of unstable atheroma, with particular characteristics: the former is defined by the presence of a thrombus overlying a thin interrupted fibrous cap with a well-represented lipid-rich necrotic core; plaque erosion consists of an area of endothelial denudation overlying a thick unbroken fibrous cap with a great number of smooth muscle cells. Platelets are activated by the exposed subendothelial collagen, resulting in the formation of a platelet-rich thrombus.

### 2.2 Coronary Non-Atherosclerotic Causes of MINOCA

#### 2.2.1 Coronary Embolism or *In-Situ* Thrombosis

Coronary thrombosis or embolism may cause MINOCA if the microcirculation is involved, with or without a hypercoagulable state. Diagnostic testing for inherited coagulopathies in patients with MINOCA should be performed when information from the patient’s personal history and family history could raise clinical concern. Coronary emboli may occur in the context of the above thrombophilic disorders or other predisposing hypercoagulable states such as atrial fibrillation and valvular heart disease. Emboli may arise from non-thrombotic sources also including valvular vegetations or calcifications, iatrogenic air emboli or cardiac tumors (e.g., papillary fibroelastoma or myxoma) [13, 23]. These different etiologies recognize the same pathogenetic mechanism, that is blockage of the microcirculation, which in turn leads to a pro-inflammatory state and to platelet activation by reiterating the pro-thrombotic stimulus, and additionally, a component of reactive vasoconstriction can be added [13, 23].

#### 2.2.2 Coronary Artery Spasm

Coronary artery spasm is a prevalent cause of MINOCA, according to recent literature data it represents about 30% of cases of MINOCA. It is characterized by an intense vasoconstriction of an artery within the coronary epicardial arterial circulation. This constriction can be either focal or diffuse, involving more than 90% of the artery’s diameter, leading to compromised myocardial blood flow [24]. The underlying mechanism of this spasm involves hyperactivity of smooth muscle cells in the vascular wall, which can be triggered by various endogenous or exogenous stimuli. For instance, substances like methamphetamine and cocaine have been known to induce spasms [25]. Coronary artery spasm commonly manifests as transient ischemia, which is the underlying cause of Prinzmetal’s angina [26]. However, in some cases, it can result in more prolonged spasms and persistent ischemia, leading to AMI [27]. It has been observed that Asian individuals have a higher risk of coronary spasm compared to individuals of white race [28]. Furthermore, recent research has demonstrated that particulate matter with a diameter of 2.5 micrometers or smaller, a component of air pollution, is an independent risk factor for the development of coronary spasm and MINOCA [29].

#### 2.2.3 Microvascular Dysfunction

Microcirculatory dysfunction (CMD) is responsible for approximately 20–30% of MINOCA cases [8, 30]. The diagnosis of microcirculatory dysfunction can be made using both invasive and non-invasive methods. Non-invasive diagnostic methods include CMR and positron emission tomography (PET). CMD is characterized by homogenous circumferential inducible ischemia, localized mainly in the subendocardial layer of the myocardium well identifiable with using 3-T CMR with quantitative perfusion [31]. Through myocardial PET it is possible to study the coronary microcirculation and evaluate its functionality, specifically by quantifying reductions in hyperemic myocardial blood flow (MBF) and myocardial flow reserve (MFR) [32]. Invasive evaluation should involve assessing both microvascular vasodilatory and vasoconstrictive responses. Microvascular vasoconstrictive responses could be evaluated using a provocative stimulus, either pharmacological (acetylcholine or ergonovine) or non-pharmacological (hyperventilation ± tris(hydroxymethyl)aminomethane (TRIS) buffer infusion or cold pressor testing). The vasodilatory capacity can be estimated using the coronary flow reserve (CFR), which is the ratio between the maximum hyperemic coronary blood flow velocity and the baseline flow velocity achieved through adenosine infusion [33]. Another parameter is the index of microcirculatory resistance (IMR), which is defined as the product of distal coronary pressure and the mean transit time of a saline bolus through a coronary artery during maximal hyperemia [34]. Under physiological conditions the IMR is <25. An IMR ≥25 indicates increased resistance to microvascular and microcirculatory dysfunction. An IMR value >40 after primary coronary angioplasty is associated with a higher incidence of major cardiovascular events at 30 days, the presence of a larger infarct area, and an increased risk of microvascular obstruction [35].

#### 2.2.4 Spontaneous Coronary Artery Dissection 

Spontaneous coronary artery dissection (SCAD) is defined as an ‘epicardial coronary artery dissection that is not associated with atherosclerosis or trauma and is not iatrogenic’. It is a common cause of AMI among women <50 years of age. SCAD is estimated to occur in 1–4% of patients with acute coronary syndromes. SCAD is caused by a separation of the media and intimal tunica with intramural hematoma protrusion into the vascular lumen [13]. It may exist as a basic intrinsic vascular disease to which precipitating factors associated with catecholamine release may be added. Extreme physical exertion, emotional stress, and sympathomimetic medications could all be triggering factors. The substantial correlation between SCAD and other vascular diseases, such as fibromuscular dysplasia, supports this notion [36]. Collagen vascular diseases like Marfan, Ehlers-Danlos and Alport syndromes, as well as inflammatory conditions like systemic lupus erythematosus, celiac disease, sarcoidosis, and inflammatory bowel disease, are also linked to SCAD. Notably, SCAD has been described in all phases of childbirth [37]. The majority of SCAD patients exhibit high serial biomarkers and electrocardiogram (ECG) results compatible with AMI as well as chest pain or similar symptoms. Sudden cardiac arrest, cardiogenic shock, and ventricular arrhythmias are further symptoms of SCAD. The final diagnosis could require intravascular imaging demonstrating the absence of significant atherosclerosis and the presence of dissection and intramural hematoma [38].

#### 2.2.5 Supply Demand Mismatch

This is a broad category that encompasses different pathophysiological processes (such as coronary spasm and thrombosis) as well as additional systemic disorders that cause a mismatch between supply and demand (such as tachyarrhythmias, anemia, hypotension, and thyrotoxicosis) [1, 13]. When there is a reasonable etiology (such as tachycardia, anemia, or hypotension) and there are no clinical or diagnostic modalities that would otherwise support a different diagnosis, a type 2 myocardial infarction is diagnosed in patients with MINOCA [39]. One of the frequent causes of type-2 myocardial infarction is tachyarrhythmia-associated AMI [40]. The treatment or reversal of the initiating cause would take precedence in the management of a MINOCA event caused by a supply-demand mismatch.

## 3. Management Strategies for MINOCA

**Fig. 2.**
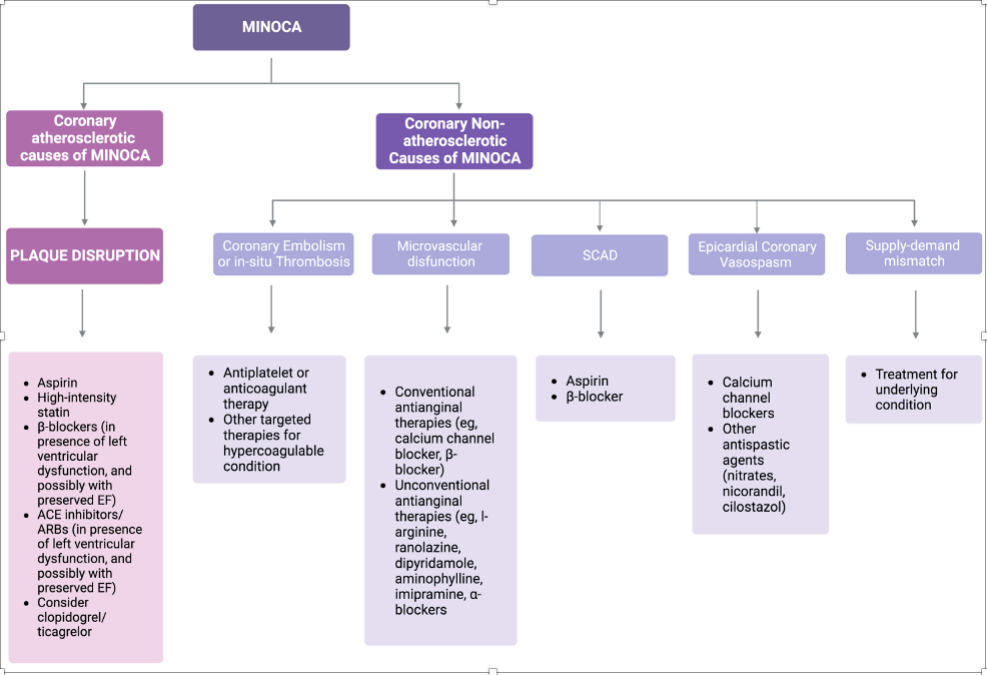
**MINOCA suggested therapeutic approaches according to different etiologies**. SCAD, spontaneous coronary artery dissection; MINOCA, myocardial infarction with non-obstructive coronary arteries; EF, ejection fraction; ACE, angiotensin-converting enzyme; ARBs, angiotensin receptor blockers.

The treatment recommendations in current guidelines are based mainly on expert opinions. Therefore, management strategies of these patients should focus on the acute treatment of any emergencies related to acute coronary syndrome [41]; otherwise, this syndrome should be considered a working diagnosis requiring a step-by-step diagnostic algorithm based on the patient’s clinical features and on the results of the instrumental investigations carried out. Once a possible responsible mechanism at the basis of the acute event has been recognized, a specific therapy is essential in addition to a generic cardioprotective therapy (Fig. 2). Important evidence is that except in SCAD, long-term low-dose aspirin is recommended for secondary prevention after MINOCA, as sustained in recent consensus documents [13].

## 4. Cardioprotective Therapies

Atherothrombosis, as previously discussed, does not play a well-defined role in all cases of MINOCA, so the value of these therapies is uncertain. Therefore, secondary preventative therapies should be considered individually for these patients. Lindahl *et al*. [39] performed a propensity analysis on 9138 MINOCA patients enrolled in the SWEDEHEART registry, analyzing the role of cardioprotective therapies. According to their results, statins, ACE inhibitors/ARBs, β-blockers, significantly reduced the composite of all-cause mortality or hospitalization for reinfarction, heart failure, or stroke at 4 years. On the other hand, the use of dual antiplatelet therapy (DAPT) was not associated with a reduced event rate, even if the entire MINOCA cohort was analyzed without distinction between those with confirmed plaque rupture or erosion and those with other etiologies [27].

### 4.1 Cause-Specific Therapies

#### 4.1.1 Plaque Disruption

In MINOCA events caused by plaque disruption cardioprotective therapies according to AMI guidelines should be prescribed [33, 34, 35], since atherothrombosis is primarily involved in pathogenesis. One of the remaining questions regarding the management of these patients is about the use of DAPT in case of a small plaque rupture in a non-significant stenosis and without overlying thrombus. The recent EROSION study was a pilot study that analyzed the role of DAPT without stenting in patients with an MI, secondary to plaque erosion documented by OCT [42]. The study showed a significant reduction in thrombus volume at one month follow-up for patients in DAPT therapy (aspirin and ticagrelor), and after 1 year 92.5% of patients in DAPT didn’t report any major adverse cardiovascular events. Therefore, this study offered encouraging data on DAPT in MINOCA with plaque disruption but further confirmation from prospective, randomized clinical trials are needed [43].

#### 4.1.2 Coronary Embolism or *In-Situ* Thrombosis

It is still debatable whether long-term anticoagulant or antiplatelet therapies are required in this group of MINOCA patients. Specific hypercoagulable states could be treated with appropriate therapies. For example, Thrombotic Thrombocytopenic Purpura (TTP) patients need plasmapheresis, with possible adjunctive treatments including steroids and rituximab [13]. Other hypercoagulable conditions require targeted therapies and a shared diagnostic and therapeutic management with a hematologist [13].

#### 4.1.3 Epicardial Coronary Vasospasm 

Calcium channel blockers are considered the keystone therapy for patients with coronary spasm. Indeed, calcium channel blockers have been demonstrated to improve angina symptoms and prognosis in this patient population [44]. For patients with refractory vasospastic angina, the use of two calcium channel blockers that act on different receptors has been shown to improve symptoms [26]. In addition to calcium channel blockers, other medications have demonstrated effectiveness in alleviating symptoms of coronary spasm. These include nitrates, nicorandil, cilostazol, and pioglitazone [45, 46, 47, 48, 49, 50, 51, 52, 53, 54]. On the other hand, the use of beta-blockers should be avoided as they can predispose individuals to episodes of vasospastic angina [55] and antiplatelet therapies have not been demonstrated to improve symptoms and/or prognosis [56].

#### 4.1.4 Coronary Microvascular Dysfunction 

The therapeutic management of patients with microvascular dysfunction is more debated compared to other forms of MINOCA. Indeed, many conventional vasodilator agents are less effective on the microvasculature than on large epicardial vessels [40]. Among conventional antianginal medications, beta-blockers and calcium channel blockers have been shown to alleviate symptoms [57]. Additionally, small studies have demonstrated the benefit of other drugs such as dipyridamole, ranolazine (due to their microvascular vasodilatory effect), imipramine, aminophylline (for their analgesic effect), L-arginine and statins (for their endothelial stabilization effect) [58]. However, further studies are needed to establish optimal management and treatment strategies for this subgroup of patients with MINOCA.

#### 4.1.5 SCAD

In terms of revascularization strategy, a conservative approach should be the preferred strategy, except for very high-risk patients [59]. In fact, it was observed that coronary segments with SCAD repair spontaneously, and revascularization is associated with a risk of dissection propagation.

In terms of pharmacological therapy, patients with SCAD should be treated with aspirin and beta-blockers [60]. SCAD survivors taking beta-blockers had a decreased risk of recurrences, according to data from a large cohort [61]. The use of a combination of anticoagulation and DAPT should be avoided since they might enhance the likelihood of bleeding and the spread of the hematoma/false lumen [62]. Indeed, in large retrospective registries, DAPT has been associated with less favourable clinical outcomes as compared to aspirin alone [63].

Finally, depending on the patient’s specific risk factors (such as dyslipidaemia) and on the left ventricular ejection fraction, statins and/or heart failure drugs can be added [64].

## 5. Conclusions

MINOCA is a distinct clinical diagnosis with many different pathophysiological causes. This aspect greatly complicates their management in clinical practice and makes it difficult to extrapolate meaningful data from clinical trials conducted in AMI patients.

Currently, after excluding other potential causes for troponin elevation, the best assessment for individuals with a diagnosis of MINOCA should focus on identifying the specific mechanism for each patient, so that individualized therapy can be employed.

Randomized clinical trials are required to assess the effectiveness of novel or conventional secondary prevention strategies to improve short- and long-term clinical outcomes in this patient population.
